# The updated evidence of pirfenidone treated silicosis based on network pharmacology, molecular docking and experimental validation

**DOI:** 10.3389/fmed.2025.1573241

**Published:** 2025-05-21

**Authors:** Sirong Chang, Shengpeng Wen, Wenyue Zhang, Huning Zhang, Yi Guo, Qiushi Wang, Xiaokun Hu, Zhihong Liu, Yue Sun, Anning Yang

**Affiliations:** ^1^School of Public Health, General Hospital of Ningxia Medical University, Ningxia Medical University, Yinchuan, Ningxia, China; ^2^Key Laboratory of Metabolic Cardiovascular Disease, Ningxia Medical University, Yinchuan, Ningxia, China

**Keywords:** silicosis, pirfenidone, network pharmacology, molecular docking, therapy

## Abstract

**Objective:**

Silicosis remains a debilitating occupational lung disease with limited therapeutic options, despite emerging evidence supporting pirfenidone’s (PFD) anti-fibrotic efficacy in clinical practice. However, the molecular circuitry governing PFD’s therapeutic actions in silicosis remains incompletely mapped, hindering mechanism-driven therapeutic optimization. To bridge this knowledge gap, we executed network pharmacology to replenish its molecular mechanisms and potential therapeutic targets.

**Materials and methods:**

We replicated a silicosis C57BL6/J mouse model and evaluated inflammation and fibrosis using HE, Masson, and Sirius Red staining assays. The expression of fibrotic markers *α*-SMA and Fibronectin were determined by immunofluorescence assay. Network pharmacology and molecular docking were used to predict potential therapeutic mechanisms and targets. Quantitative reverse transcription polymerase chain reaction (qRT-PCR) and immunofluorescence experiments were verified as the key predicted targets.

**Results:**

PFD alleviated the level of inflammation and collagen deposition and fibrotic markers *α*-SMA and Fibronectin expression in silicosis lung. Network pharmacology analysis predicted three potential target proteins, including TNF, MMP9, and NF-κB1, as well as ten possible signaling pathways. Molecular docking showed a good binding activity between PFD and hub genes. qRT-PCR and immunofluorescence confirmed that PFD inhibited TNF, MMP9, and NF-κB activation. Additionally, we found increased expression of TLR2, a key upstream gene of NF-κB.

**Conclusion:**

In conclusion, we identified TNF, MMP9, NF-κB1 and TLR2, that contribute to the therapeutic effects of PFD in silicosis. Mechanistically, PFD appears to mitigate silicosis pathogenesis through suppression of epithelial TLR2/NF-κB pathway activation.

## 1 Introduction

Silicosis is a global public health issue, associated with extensive exposure in industries such as mining, construction, manufacturing, and agriculture, particularly in developing countries, where the incidence of silicosis is high due to inadequate industrial environments and protective measures (1). According to the Global Burden of Disease (GBD) data, from 1990 to 2019, the absolute number of incidence, prevalence, and Disability Adjusted Life Year (DALY) of pneumoconiosis worldwide have shown an upward trend, while the number of deaths has decreased (2). Silicosis is an occupational lung disease caused by long-term inhalation of dust containing > 10% free crystalline silica (3). The disease development process is characterized by the formation of nodular, progressive, irreversible, and fatal pulmonary fibrosis, leading to impaired lung function and respiratory disorders (4, 5). Current research aims to understand the pathogenesis of silicosis to identify preventive or therapeutic anti-fibrotic drugs; however, studies on silica-induced pulmonary fibrosis lags clinical needs (6, 7). As symptomatic treatment remains the primary clinical approach, exploring more effective drugs and clarifying their therapeutic mechanisms is essential for delaying or improving silicosis (7).

Pirfenidone (PFD) is an approved pharmaceutical agent for treating idiopathic pulmonary fibrosis (IPF) due to its anti-fibrotic, anti-inflammatory, and antioxidant properties (8, 9). Given the pathological similarities between silicosis and IPF, particularly in pulmonary fibrosis formation, the therapeutic potential of PFD for silicosis is gaining attention. Studies indicate that PFD can reduce inflammatory cell infiltration, inhibit inflammatory factor production, and decrease Collagen I and Fibronectin accumulation. Notably, in early-stage silicosis, PFD effectively alleviates lung dysfunction, pulmonary hypertension, inflammation, and fibrosis (10). Additionally, PFD enhances lung function in silicosis models by inhibiting IL-17A secretion, suggesting IL-17A may be a key therapeutic target (11). Phase III clinical trials have shown PFD’s efficacy in IPF, including a slowdown in lung function decline and reduced mortality risk (12, 13). Despite these advances, research on PFD’s application and mechanism in silicosis remains limited. Therefore, further investigation into PFD’s mechanisms in treating silicosis is essential for understanding its potential effects and identifying new therapeutic targets, ultimately leading to more effective treatment strategies.

**Figure 1:**
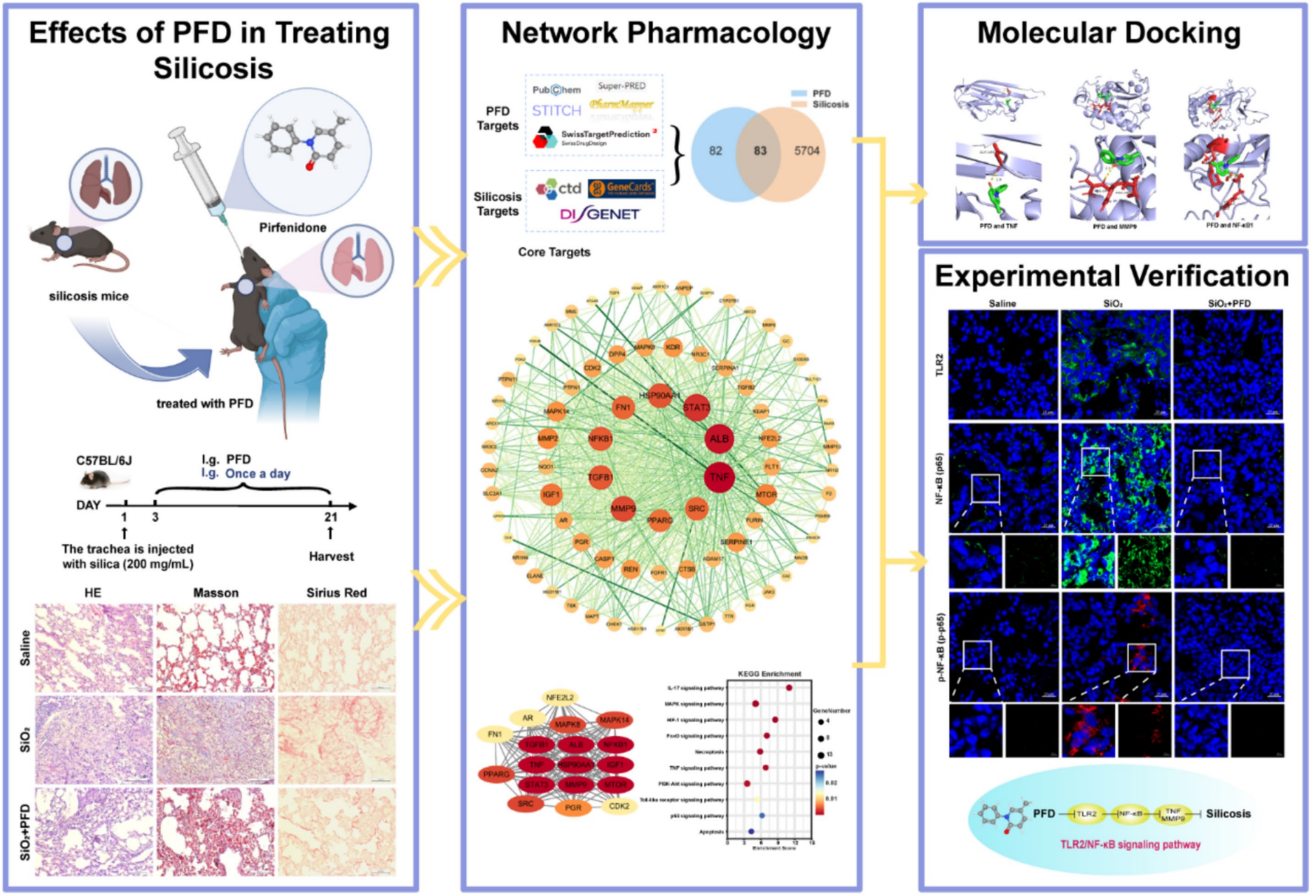
Technical strategy of the research. Select schematics were generated using BioRender (https://BioRender.com).

With the rapid advancement of bioinformatics and systems biology, network pharmacology (14), as a powerful means, has been widely applied to reveal the complex mechanisms of drug action. Network pharmacology leverages interaction databases, enrichment tools, and systems-level modeling to connect drugs with disease genes, providing hypotheses for mechanisms of action. This study has two main objectives: (1) to explore the potential targets and mechanisms of PFD against silicosis using network pharmacology and molecular docking, and (2) to validate PFD’s antifibrotic effects and related pathways, elucidating its molecular mechanism (Figure 1).

## 2 Materials and methods

### 2.1 Instruments and reagents

Silica (>99% purity, 0.5 to 10 μm) was purchased from Sigma, USA. The A549 cell line was gained from Zhongqiaoxinzhou, China. C29 was purchased from MEDCHEMEXPRESS LLC, USA. High-glucose medium was obtained from Gibco, USA. HE, Masson, and Sirius Red staining kits, PFD, penicillin–streptomycin, as well as DAPI and anti-fluorescence quencher, were obtained from Solarbio, China. 4% paraformaldehyde solution were sourced from Saint Biotechnology, China, and phosphate-buffered saline (PBS) from Invigentech, USA. Primary antibodies, including anti-TNF, anti-NF-κB (p65), and anti-p-NF-κB (p-p65), were sourced from Affinity Bioscience, USA; anti-MMP9 from Proteintech, China; anti-*α*-SMA from Abcam, UK; anti-Fibronectin and anti-TLR2 from Santa Cruz Biotechnology, USA. Secondary antibodies Alexa Fluor® 488 and Alexa Fluor® 647 were purchased from Abcam, UK. Total RNA Extraction Kit were obtained from TIANGEN, China. The PrimeScript RT Master Mix and the TB Green Premix Ex Taq II were purchased from TaKaRa, Japan. QuantStudio 5 Real Time PCR system was sourced from Thermo Fisher Scientific, USA. The LSM800 laser confocal microscope was obtained from Zeiss, Germany.

### 2.2 Establishment of mouse silicosis model

C57BL/6 J mice aged 6–8 weeks were sourced from the Experimental Animal Research Center of Ningxia Medical University. After 1 week of acclimatization, they were randomly divided into three groups: Saline control group, SiO_2_ model group, and SiO_2_ + PFD treatment group, with 6 mice each. Mice in the control group received saline injections, while those in the model and treatment groups were injected with 0.1 mL of a 200 mg/mL SiO₂ suspension. Forty-eight hours post-injection, mice in the treatment group were given PFD (300 mg/kg, dissolved in saline) (15) via intragastric gavage once daily, continuing until the conclusion of the experiment on day 21. To minimize handling-related stress bias, all groups (including the model group) underwent identical handling procedures. The model group received sham gavage (brief oral insertion of an empty gavage needle without substance delivery) at the same frequency as the treatment groups.

### 2.3 Histological

Lung tissues were fixed in 4% paraformaldehyde for 48 h, then embedded and sectioned at a thickness of 5 μm. Sections were stained with H&E, Masson, and Sirius Red according to the manufacturer’s instructions, and then images were captured.

### 2.4 Immunofluorescence

Frozen lung sections were warmed to room temperature in a moist chamber, fixed with 4% paraformaldehyde, and permeabilized. After blocking, sections were incubated overnight with the primary antibody at 4°C. The following day, they were warmed again, and a fluorescent secondary antibody was applied and incubated at 37°C for 1 h. After washing with PBS, DAPI was used to stain the nuclei. Sections were then covered with an anti-fluorescence quencher and examined under a laser confocal microscope.

### 2.5 Construction of the PFD drug target library

The molecular structure and SMILE notation of PFD were identified by searching “Pirfenidone” in the PubChem database.^1^ Subsequently, active targets of PFD were collected from the PharmMapper,^2^ SwissTargetPrediction,^3^ SuperPred,^4^ and STITCH ^5^databases. The SDF file of PFD was input into PharmMapper and SwissTargetPrediction databases to gather targets, while the SMILE string was uploaded to STITCH for targets recognition. PharmMapper database predicts proteins based on the pharmacophore model, applying a filter for “Human Protein Targets Only and z-score>0.” The SwissTargetPrediction results was filtered for “probability>0” to obtain relevant target names. SuperPred database (Probability≥50%) and in STITCH, the target information for PFD was retrieved using “*Homo sapiens*” as the qualification condition and a “combined score ≥ 0.4” as the selection threshold. The resulting target names and Uniprot IDs were converted to standard gene names using the Uniprot ^6^database, eliminating duplicate.

### 2.6 Construction of the silicosis disease target library

The keywords “silicosis” were used to search for targets related to silica-induced pulmonary fibrosis in the GeneCards (https://www.genecards.org/), DisGeNET (https://www.disgenet.org/), and CTD (http://ctdbase.org/) databases. Data with CTD scores higher than the average were selected, and the search results from the three databases were integrated, removing duplicates to construct the disease target library.

### 2.7 Analysis of potential targets for PFD anti-silicosis and PPI network construction

A Venn diagram was used to identify common potential targets between PFD drug targets and silicosis disease targets, with the intersecting section considered potential targets for PFD treatment of silicosis. This targets were imported into the STRING platform (see text footnote 7) with a minimum interaction score set to “medium confidence = 0.4.” Nodes without interaction links were hidden, and other parameters were kept unchanged to obtain protein–protein interaction (PPI) relationships. The data file was then exported and imported into Cytoscape 3.10.2 for visualization analysis. Parameters were adjusted so that node size and color intensity reflected degree value, while edge thickness indicated binding rate score, resulting in a PPI network diagram.

### 2.8 GO and KEGG enrichment analysis of PFD anti-silicosis potential targets

The DAVID^8^ database was used to annotate potential action targets by entering the gene symbols, selecting “OFFICIAL_GENE_SYMBOL” as the Identifier, “*Homo sapiens*” as the species, and “Gene List” as the list type. Gene Ontology (GO) Enrichment Analysis and Kyoto Encyclopedia of Genes and Genomes (KEGG) enrichment analysis were performed. GO functional analysis examined the main biological functions of PFD treatment for silicosis, including potential biological processes (BP), molecular functions (MF), and cellular components (CC). KEGG pathway analysis assessed the primary signaling pathways associated with PFD treatment. Results were visualized using the OE platform^9^ by creating bubble charts.

### 2.9 Molecular docking

The SDF file of PFD was downloaded from the PubChem database and converted to PDB format using OpenBabel 2.4.1. The PDB IDs with the lowest Resolution values for core targets were selected from the UniProt database, and the corresponding protein structure PDB files were downloaded from PDB^10^ database. Molecular docking was performed using AutoDock Vina 1.5.7, and the results were visualized with PyMOL.

### 2.10 Quantitative reverse transcription polymerase chain reaction (qRT-PCR)

Lung tissue was weighed and homogenized in RZ lysis buffer. Total RNA was extracted from lung tissue following the instructions of the Total RNA Extraction Kit. The reverse transcription system was constructed using the PrimeScript RT Master Mix. Real-time fluorescent quantitative PCR was performed using the TB Green Premix Ex Taq II. The primer sequence were as follows^11^: TNF (NCBI Gene ID: 21926) (F: 5’-CCCTCACACTCAGATCATCTTCT-3′; R: 5’-GCTACGACGTGGGCTACAG-3′), MMP9 (NCBI Gene ID: 17395) (F: 5’-CTGGACAGCCAGACACTAAAG-3′; R: 5’-CTCGC GGCAAGTCTTCAGAG-3′), TLR2 (NCBI Gene ID: 24088) (F: 5’-GCAAACGCTGTTCTGCTCAG-3′; R: 5’-AGGCGTCTCCCTC TATTGTATT-3′), NF-κB1 (NCBI Gene ID: 18033) (F: 5’-ATGGCAGACGATGATCCCTAC-3′; R: 5’-TGTTGACAGTG GTATTTCTGGTG-3′), GAPDH (NCBI Gene ID: 14433) (F: 5’-AGGTCGGTGTGAACGGATTTG-3′; R: 5’-TGTAGACCATGTA GTTGAGGTCA-3′). The relative expression was calculated using the 2^-ΔΔct^.

### 2.11 Cell culture

A549 cells were cultured in high-glucose medium containing 1% penicillin–streptomycin and 10% fetal bovine serum at 37°C with 5% CO₂. Cells were treated with 100 μg/mL SiO₂ for 24 h to establish a silicosis epithelial cell injury model. The treatment groups received PFD (50 μM, dissolved in PBS) (16) and C29 (50 μM, a specific TLR2 inhibitor, dissolved in PBS with 10% DMSO) (17).

### 2.12 Statistical analysis

Experimental results are expressed as the mean ± standard deviation. Statistical analysis was conducted using SPSS 24.0. A one-way analysis of variance (ANOVA) was used for comparisons among multiple groups, with post-hoc multiple comparisons performed using Tukey’s test. A *p* < 0.05 was considered statistically significant.

## 3 Results

### 3.1 PFD alleviates silica-induced pulmonary fibrosis in mice

**Figure 2:**
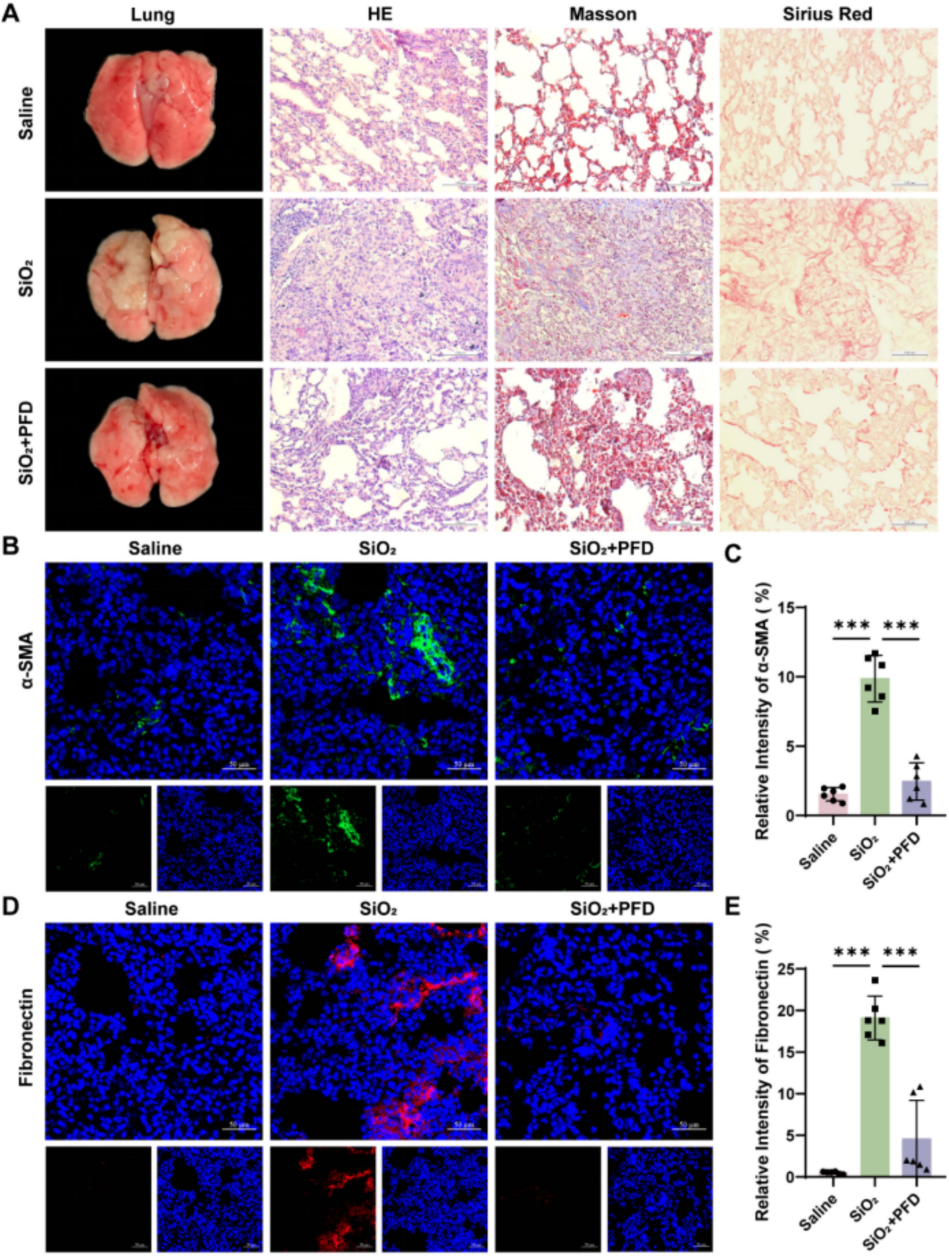
PFD alleviates silica-induced pulmonary fibrosis in mice. **(A)** Lung appearance and pathological staining such as HE, Masson and Sirius Red. Scale bars, 100 μm. **(B,C)** Fluorescence expression and quantification of *α*-SMA. **(D,E)** Fluorescence expression and quantification of Fibronectin. Scale bars, 50 μm. *p < 0.05, **p < 0.01, ***p < 0.001 (mean ± SD).

After establishing a silicosis mouse model, we assessed the efficacy of PFD treatment by examining the gross pulmonary morphology and pathological staining, including HE, Masson, and Sirius Red. The staining results indicated that, compared to the control group, the model group mice exhibited significant inflammatory cell infiltration and substantial collagen deposition in lung tissue, along with characteristic silicon nodules. HE staining revealed that PFD reduced inflammatory cell infiltration and silicon nodule formation in silica-damaged lung tissue. Masson and Sirius Red Staining further demonstrated that PFD could decrease collagen deposition in the interstitial spaces of lung tissue (Figure 2A). Furthermore, immunofluorescence assay confirmed that PFD markedly reduced silica-induced fluorescence expression of fibrosis-related markers *α*-smooth muscle actin (α-SMA) and Fibronectin in mouse lung tissue, decreasing levels by approximately fourfold for both markers (Figures 2B–E). In summary, our results suggested that PFD alleviates collagen deposition and fibrosis in the lung tissue of silicosis mice *in vivo*.

### 3.2 Network pharmacology reveals the potential mechanisms of PFD in combating silicosis

**Figure 3:**
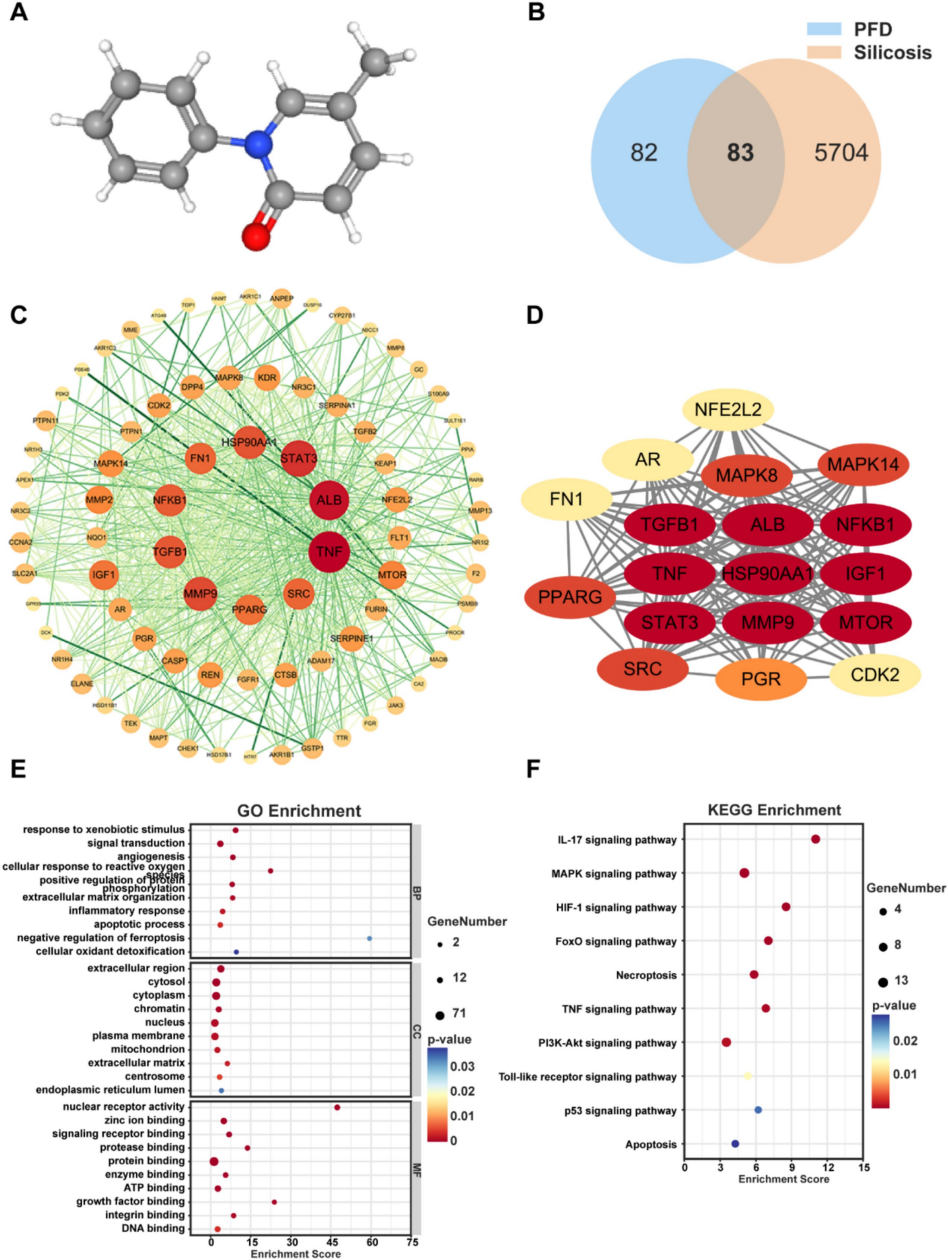
Network pharmacology reveals the molecular mechanism of PFD for silicosis. **(A)** 3D molecular structure of PFD. **(B)** Venn diagram of common targets of PFD and silicosis. **(C)** PPI network of intersection targets. **(D)** GO enrichment map of hub gene. **(F)** KEGG pathway analysis of intersection genes.

We initially obtained the molecular structure of PFD from the PubChem database (Figure 3A). Subsequently, 156 PFD targets were screened from the PharmMapper, Swiss Target Prediction, SuperPred, and STITCH databases and identified 5,787 Subsequently, 156 PFD targets were screened from the GeneCards, DisGeNET, and CTD databases. Then, 83 overlapping targets were obtained by integrating these drug and disease targets and taking their intersection, which is illustrated by a Venn diagram (Figure 3B).

Next, we constructed a PPI network using the STRING database and analyzed the topological properties of the network nodes, including degree and betweenness centrality, using Cytoscape software. This resulted in a visualized and optimized PPI network diagram (Figure 3C). Network analysis identified 18 core targets for PFD treatment of silicosis, highlighting their interactions in a specific PPI network diagram (Figure 3D). Notably, the top ten targets based on degree values were Tumor Necrosis Factor (TNF), Albumin (ALB), Signal Transducer and Activator of Transcription 3 (STAT3), Matrix Metalloproteinase 9 (MMP9), Heat Shock Protein 90 Alpha Family Class A Member 1 (HSP90AA1), Transforming Growth Factor Beta 1 (TGFB1), Nuclear Factor Kappa B Subunit 1 (NF-κB1), Fibronectin 1 (FN1), SRC Proto-Oncogene, Non-Receptor Tyrosine Kinase (SRC), and Peroxisome Proliferator-Activated Receptor Gamma (PPARG). Current research widely recognizes that these above genes encode proteins which involve fibrotic regulated processes, such as signal transduction, inflammatory responses, extracellular matrix remodeling, and immune regulation.

The PPI network constructed using Cytoscape software visually represents complex interactions among the potential therapeutic targets identified in this study (18). Node size and color indicate degree values; larger nodes signify more active targets. There were 433 common targets according to GO enrichment data, including BP (308 items), CC (44 items), and MF (81 items). Key enriched results show that PFD was involved in responses to external stimuli, signal transduction, angiogenesis, reactions to reactive oxygen species, positive regulation of protein phosphorylation, extracellular matrix organization, inflammatory responses, apoptotic processes, negative regulation of ferroptosis, and cellular oxidative detoxification. Additionally, PFD affected fibrosis through molecular functions such as nuclear receptor activity, zinc ion binding, and signal receptor binding. PFD target proteins were in the extracellular region, cytoplasm, chromatin, nucleus, plasma membrane, and mitochondria (Figure 3E).

Using KEGG enrichment analysis, we identified 105 pathways associated with PFD targets, from which we selected 10 significant pathways relevant to PFD’s resistance to silica-induced pulmonary fibrosis. Several of these pathways overlap with the biological processes highlighted in the GO enrichment data, underscoring their relevance to fibrosis. For example, the inflammatory and immune-related pathways, including the Interleukin-17 (IL-17) signaling pathway, the Mitogen-Activated Protein Kinase (MAPK) signaling pathway, the Tumor Necrosis Factor (TNF) signaling pathway, and the Toll-like receptor signaling pathway, are closely linked to the inflammatory processes involved in fibrosis. Additionally, cellular necrosis, apoptosis, and oxidative stress responses—critical components in the pathogenesis of silicosis - were highlighted through these pathways. Notably, the FoxO signaling pathway, the Phosphatidylinositol 3-Kinase-Protein Kinase B (PI3K-Akt) signaling pathway, and the p53 signaling pathway are crucial for regulating cell apoptosis, cell cycle control, and antioxidant stress responses, all of which play vital roles in fibrosis (Figure 3F). The overlap between KEGG and GO reinforces the interconnectedness of these pathways, highlighting the complex and multifaceted nature of PFD’s potential effects on silicosis.

### 3.3 Molecular docking confirms the binding energy of PFD with key molecules

**Figure 4:**
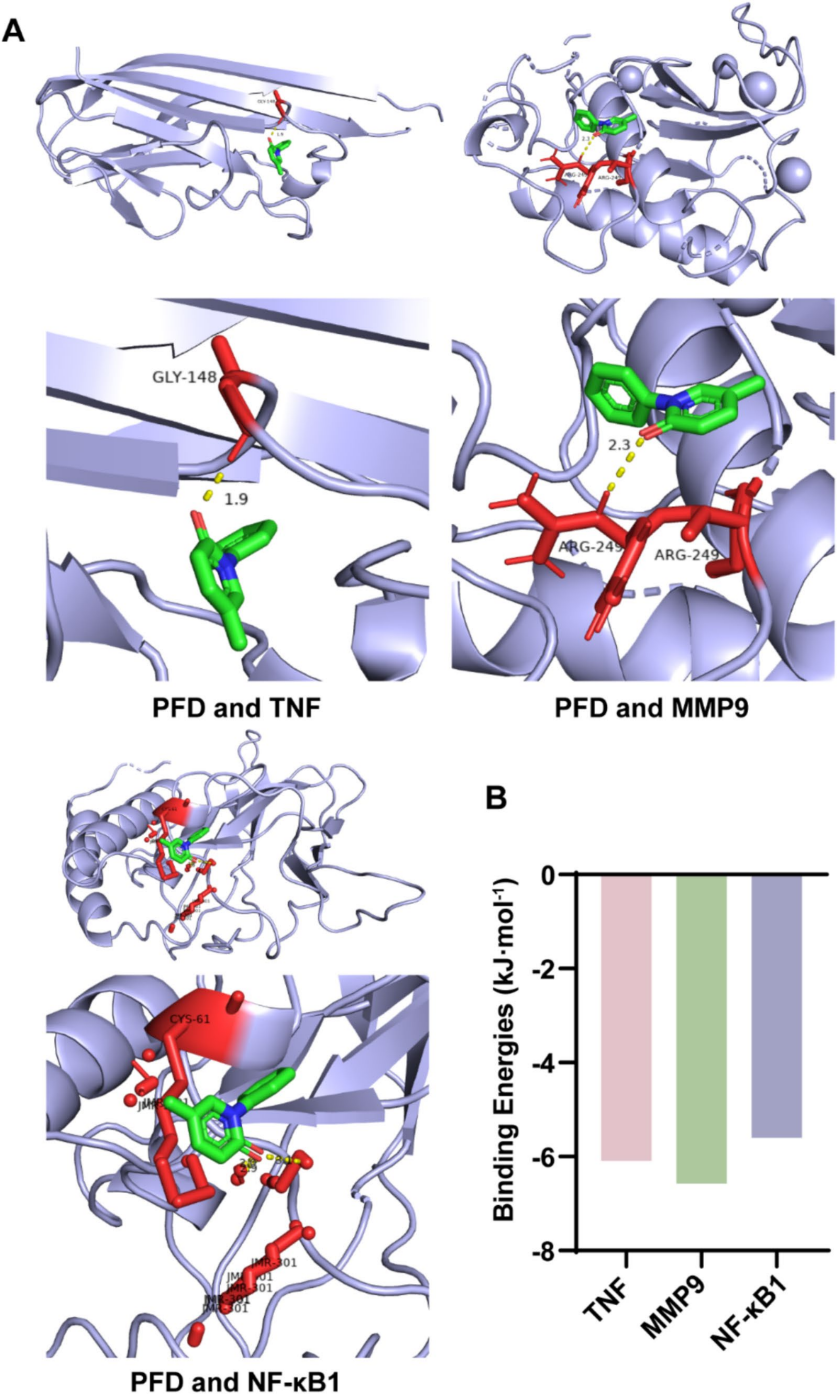
Molecular docking of PFD with key genes involved in silicosis pathology. **(A)** Molecular docking was performed to elucidate the binding mode of PFD with the proteins TNF, MMP9 and NF-κB1. **(B)** The binding energies, indicative of the molecular affinities, are depicted in a bar graph for PFD complexed with each of the key genes.

Then, we conducted molecular docking between the key targets and PFD using AutoDock Vina software to assess the binding energy. The docking energies between PFD, and TNF, MMP9, and NF-κB1 showed negative value (Figure 4A). The binding energy calculation determines the compatibility level between the small molecule and the protein and lower binding energy indicates higher stability (19). The binding energies of PFD with TNF, MMP9 and NF-κB1 were −6.09, −6.57 and −5.6 kcal/mol, respectively (Figure 4B).

### 3.4 Experimental validation of key molecules expression in silicosis

**Figure 5:**
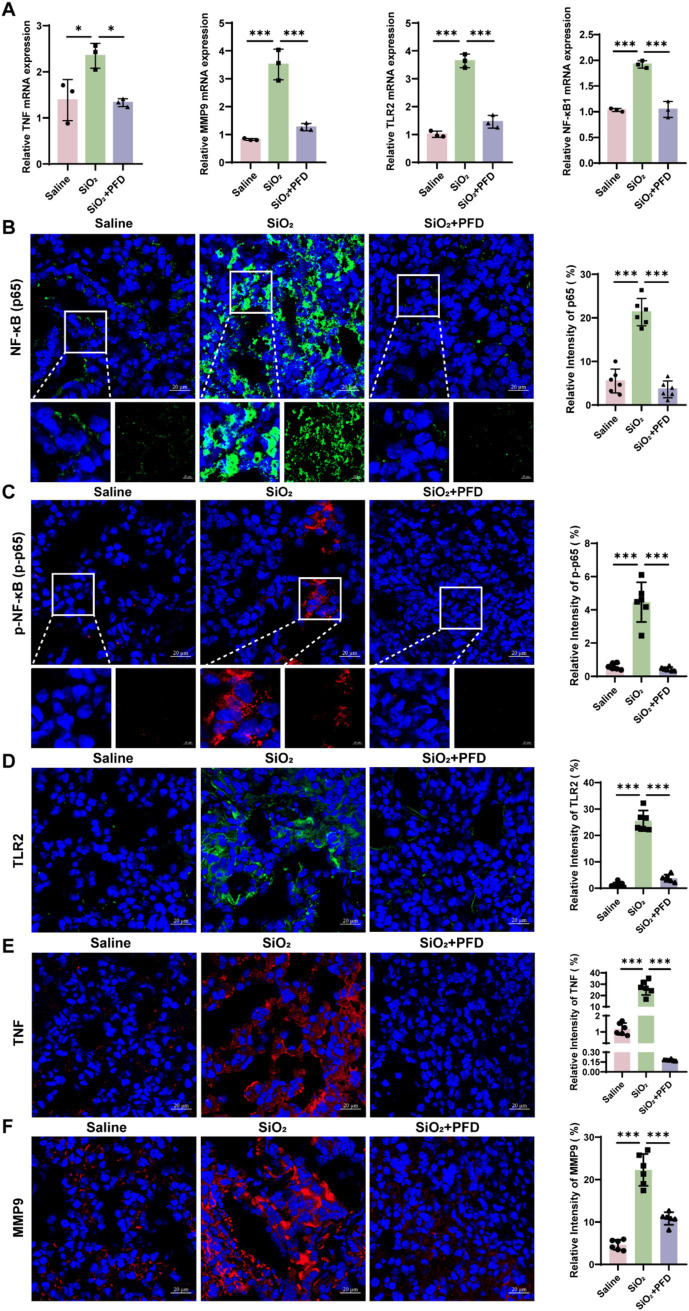
PFD alleviates silica-induced expression of TNF, MMP9, and TLR2 in mouse lung tissue, as well as the activation of NF-κB. **(A)** mRNA levels of TNF, MMP9, TLR2, and NF-κB1 across different groups, measured by qRT-PCR. **(B–F)** Immunofluorescence staining was employed to visualize and quantify the fluorescence expression of p65, p-p65, TLR2, TNF and MMP9 following PFD treatment. Scale bars, 20 μm. *p < 0.05, **p < 0.01, ***p < 0.001 (mean ± SD).

**Figure 6:**
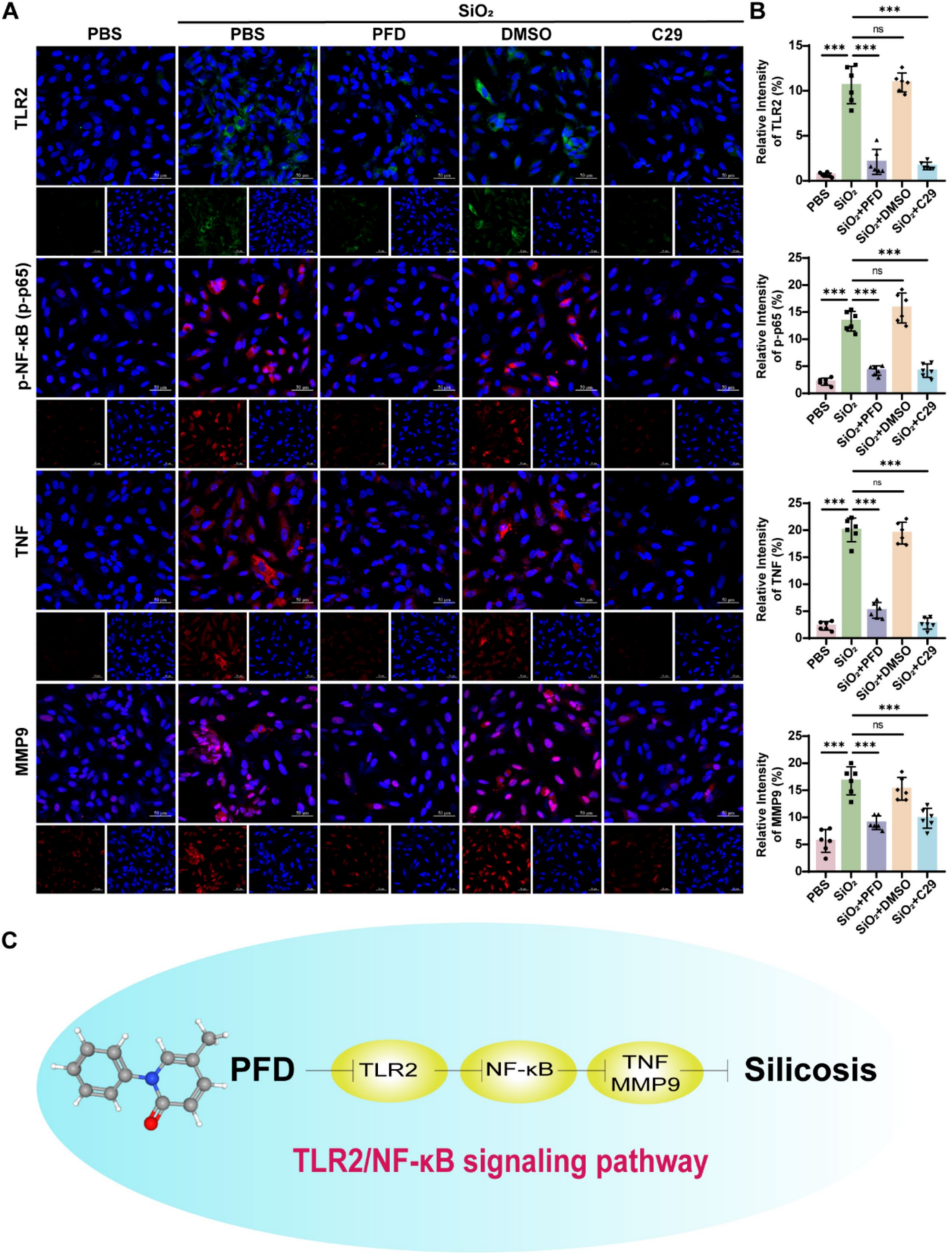
PFD inhibits silica-induced expression of TNF, MMP9, TLR2, and NF-κB activation in A549 cells. **(A,B)** Immunofluorescence staining was used to observe and quantify the fluorescence expression of TLR2, p-p65, TNF, and MMP9 in A549 cells after treatment with PFD and C29. Scale bar, 50 μm. *p < 0.05, **p < 0.01, ***p < 0.001 (mean ± SD). **(C)** PFD mediates its anti-fibrotic action through the modulation of key molecules and signaling pathways. PFD reduces inflammation and fibrosis by inhibiting the expression of crucial molecules such as TLR2, NF-κB, TNF, and MMP9. Specifically, PFD may inhibit the TLR2/NF-κB pathway, further suppressing the secretion of TNF and the activity of MMP9, thereby alleviating inflammation and extracellular matrix deposition to exert its anti-fibrotic effects.

Network pharmacology identifies TNF, MMP9, and NF-κB1 as key genes in PFD’s anti-silicosis effect. TNF, a pro-inflammatory cytokine, induces MMP9 expression and secretion (20). NF-κB plays a crucial role in immune regulation and inflammation (21). Research shows that Toll-like receptors, especially TLR2, are pivotal in initiating inflammation and TLR2 activation triggers downstream signaling, leading to NF-κB activation and an inflammatory response (22, 23). To further clarify the molecular mechanisms of PFD’s anti-silicosis effects, we performed *in vivo* and *in vitro* experiments. First, we measured the mRNA levels of core molecules in lung tissue and found that silica induced increased expression of TNF, MMP9, TLR2 and NF-κB1, while PFD effectively reversed this response (Figure 5A). NF-κB1 is a critical regulator of the NF-κB signaling pathway. Dysregulated activation of NF-κB1 leads to sustained pathway activation, which in turn promotes its own transcription and translation, resulting in elevated expression levels (24). Our data show that silica exposure significantly upregulates NF-κB1 expression, reflecting persistent activation of the NF-κB pathway. To further investigate this, we assessed the expression of p65 and phosphorylated p65 (p-p65) by immunofluorescence. Silica treatment induced a marked increase in nuclear p65 expression and upregulated p-p65, indicating persistent NF-κB pathway activation in silicosis mice. Notably, PFD treatment effectively suppressed this aberrant NF-κB activation (Figures 5B,C). Previous studies have shown that TLR2 activation significantly enhances TNF-*α* production via the NF-κB p50/p65 pathway (25). Consistent with these findings, our immunofluorescence analysis revealed a marked upregulation of TLR2 expression in the model group, which was reversed by treatment. Moreover, PFD treatment effectively attenuated the silica-induced upregulation of both TNF-α and MMP9, further underscoring its protective effects (Figures 5D–F). To further elucidate the mechanism of PFD, we conducted *in vitro* studies using A549 cells. The same as the TLR2 inhibitor C29, PFD significantly reversed the silica-induced increase in TLR2, p-p65, TNF, and MMP9 expression in A549 cells (Figures 6A,B).

## 4 Discussion

Silicosis, as a severe occupational hazard disease, is a significant public health issue. The treatment of silicosis remains inadequate and mainly therapy strategies are still controlling symptoms. PFD with anti-fibrotic, anti-inflammatory, and antioxidant effects has been used for treating IPF (26–28). In our study, based on network pharmacology, we found that PFD alleviated silica-induced pulmonary fibrosis by modulating key genes (e.g., TNF, MMP9 and NF-κB1) and engaging in immune-inflammatory processes through the following thematically grouped signaling pathways: (1) inflammatory responses (Toll-like receptor, IL-17, NF-κB, and TNF pathways), (2) cellular survival/apoptosis regulation (PI3K-Akt and MAPK pathways), and (3) hypoxia/stress responses (HIF-1 pathway).

TNF a classical pro-inflammatory cytokine, stimulates fibroblasts proliferation and interacts with other inflammatory mediators, such as IL-1β, IL-6, and IL-8, to modulate immune responses and inflammatory reactions which are associated with silicosis (29–31). Overexpression of TNF-*α* result in the progression of the disease, making it a viable detection biomarker for the early-stage silicosis patients (32). Our results clearly demonstrated that PFD downregulated the level of TNF after silica stimulant.

MMP9 remodel lung fibrosis (33). In the early silicosis stages, MMP9 disrupts lung tissue and increases the infiltration of inflammatory cells and corresponding inflammatory mediators, such as TNF-*α* and other cytokines, to promote the development of pulmonary fibrosis (34, 35). In silicosis, MMP9 and TIMP1, its endogenous inhibitors are disrupted to accelerate abnormal deposition of the Extracellular Matrix (ECM) (36). MMP9 also regulates immune cell function, including macrophages and T cells (37). Moreover, MMP9 in silicosis is associated with the degree of severity and progression lung fibrosis, which is consistent with data from network pharmacology analysis. In all, PFD alleviates the level of MMP9 expression in silicosis lungs to exert its anti-silicosis fibrosis effect.

TLR2 activation has been established as a key mediator of inflammatory and immune responses through dual signaling mechanisms involving both MyD88-dependent and TRIF-dependent pathways (38). In the MyD88-dependent pathway, TLR2 activation mediates the recruitment of MyD88 to its intracellular TIR domain, subsequently triggering sequential phosphorylation of interleukin-1 receptor-associated kinases (IRAKs). This signaling cascade ultimately activates NF-κB and mitogen-activated protein kinases (MAPKs), transcriptional regulators responsible for driving pro-inflammatory cytokine production including TNF-*α*, IL-6, and IL-1β (25). NF-κB1, a transcription factor in NF-κB signaling pathway, is associated with inflammation, cell proliferation, differentiation, and apoptosis, regulate MMP9 and heme oxygenase 1 (HMOX1) (39–41). Upon activation, A p50/p65 dimer, a typical NF-κB1 forms, transfer to the nucleus to activate inflammation-related genes such as TNF-α, IL-6, and IL-1β (42). In silicosis, NF-κB1 activation induce apoptosis in alveolar epithelial cells and macrophages, exacerbating inflammation and lungs fibrosis (43, 44). Our experimental findings demonstrate that silica exposure significantly upregulated TLR2 expression and enhanced p65 activation in murine lung tissues, as evidenced by nuclear translocation of p65 and increased phosphorylation levels of nuclear p-p65. Importantly, PFD treatment effectively attenuated these silica-induced effects, suppressing both TLR2 expression and NF-κB p65 phosphorylation. This suggests that PFD may mitigate silicosis progression through modulation of TLR2-mediated NF-κB p50/p65 pathway activation, consequently reducing TNF and MMP9 production-two critical mediators of inflammatory response and extracellular matrix remodeling in pulmonary fibrosis.

The central role of alveolar epithelial cells in maintaining pulmonary homeostasis and their pathological contributions to silicosis through oxidative stress, apoptotic signaling, epithelial-mesenchymal transition (EMT), and senescence-associated secretory phenotype (SASP) formation has been increasingly recognized (45, 46). Our *in vitro* studies demonstrate that PFD exerts its anti-fibrotic effects by downregulating TLR2 expression in lung epithelial cells and inhibiting NF-κB phosphorylation, thereby reducing TNF and MMP9 secretion. This highlights PFD’s potential therapeutic mechanism in disrupting pro-inflammatory signaling and attenuating protease-mediated tissue remodeling in silicosis (Figure 6C).

## 5 Conclusion

In summary, we employed network pharmacology to investigate the main mechanisms for PFD combating silica-induced pulmonary fibrosis. We found that PFD may alleviate inflammation and fibrosis in silicosis by inhibiting the TLR2/NF-κB pathway in epithelial cells, thereby reducing the production of TNF and MMP9. This study elucidates the mechanistic basis of PFD’s anti-fibrotic action in silicosis pathogenesis.

## 6 Limitations of the study

In this study, we identified potential targets and signaling pathways for PFD treated silicosis and experimentally validated the key target proteins. Although, we have identified key proteins in the TLR2/NF-κB signaling pathway, other important targets and pathways require further investigation and confirmation. Additionally, targets outside the central network also have a significant impact on silicosis warrant, which is needed to further investigate. Finally, our study used a mouse model of silicosis, which may not fully capture the complexity of human silicosis. Differences in immune responses, fibrotic mechanisms, and genetic backgrounds between mice and humans could impact the relevance of our findings to human patients. Future research should validate these targets in human samples and assess their translational potential in more clinically relevant settings.

## Data Availability

The original contributions of this study are included in the article. For further inquiries, please contact the corresponding author. The datasets are available in online repositories (https://www.jianguoyun.com/p/DXVpGl0QoZquDRiU3vIFIAA). The following public databases were used: PubChem database (https://pubchem.ncbi.nlm.nih.gov/); PharmMapper (https://www.lilab-ecust.cn/pharmmapper/); SwissTargetPrediction database (http://SwissTargetPrediction.ch); SuperPred (https://prediction.charite.de/); STITCH (http://stitch.embl.de); UniProt database (https://www.uniprot.org/); DisGeNET database (https://www.disgenet.org/); GeneCards database (https://www.genecards.org/); CTD database (https://ctdbase.org); STRING platform (https://www.stringdb.org/); DAVID database (https://david.ncifcrf.gov/); OE platform (https://www.oebiotech.com); RCSB PDB database (https://www.rcsb.org/).

## Footnotes

^1^ https://pubchem.ncbi.nlm.nih.gov/

^2^ https://www.lilab-ecust.cn/pharmmapper/

^3^ http://SwissTargetPrediction.ch

^4^ https://prediction.charite.de/

^5^ http://stitch.embl.de

^6^ https://www.uniprot.org/

^8^ https://david.ncifcrf.gov/summary.jsp

^9^ https://www.oebiotech.com

^10^ https://www.rcsb.org/

^11^ https://www.ncbi.nlm.nih.gov/

## References

[1] Liu X Jiang Q Wu P Han L Zhou P. Global incidence, prevalence and disease burden of silicosis: 30 years' overview and forecasted trends. BMC Public Health. (2023) 23:1366. doi: 10.1186/s12889-023-16295-2, PMID: 37461046 PMC10353232

[2] Chen S Liu M Xie F. Global and national burden and trends of mortality and disability-adjusted life years for silicosis, from 1990 to 2019: results from the global burden of disease study 2019. BMC Pulm Med. (2022) 22:240. doi: 10.1186/s12890-022-02040-9, PMID: 35729551 PMC9210623

[3] Leung CC Yu IT Chen W. Silicosis. Lancet. (2012) 379:2008–18. doi: 10.1016/s0140-6736(12)60235-9 22534002

[4] Bao R Wang Q Yu M Zeng Y Wen S Liu T. AAV9-HGF cooperating with TGF-beta/Smad inhibitor attenuates silicosis fibrosis via inhibiting ferroptosis. Biomed Pharmacother. (2023) 161:114537. doi: 10.1016/j.biopha.2023.114537, PMID: 36933378

[5] Hoy RF Chambers DC. Silica-related diseases in the modern world. Allergy. (2020) 75:2805–17. doi: 10.1111/all.14202, PMID: 31989662

[6] Krefft S Wolff J Rose C. Silicosis: an update and guide for clinicians. Clin Chest Med. (2020) 41:709–22. doi: 10.1016/j.ccm.2020.08.012, PMID: 33153689

[7] Li R Kang H Chen S. From basic research to clinical practice: considerations for treatment drugs for silicosis. Int J Mol Sci. (2023) 24:8333. doi: 10.3390/ijms24098333, PMID: 37176040 PMC10179659

[8] Martinez FJ Collard HR Pardo A Raghu G Richeldi L Selman M. Idiopathic pulmonary fibrosis. Nat Rev Dis Primers. (2017) 3:17074. doi: 10.1038/nrdp.2017.74, PMID: 29052582

[9] Taniguchi H Ebina M Kondoh Y Ogura T Azuma A Suga M. Pirfenidone in idiopathic pulmonary fibrosis. Eur Respir J. (2010) 35:821–9. doi: 10.1183/09031936.00005209, PMID: 19996196

[10] Tang Q Xing C Li M Jia Q Bo C Zhang Z. Pirfenidone ameliorates pulmonary inflammation and fibrosis in a rat silicosis model by inhibiting macrophage polarization and JAK2/STAT3 signaling pathways. Ecotoxicol Environ Saf. (2022) 244:114066. doi: 10.1016/j.ecoenv.2022.114066, PMID: 36108436

[11] Cao ZJ Liu Y Zhang Z Yang PR Li ZG Song MY. Pirfenidone ameliorates silica-induced lung inflammation and fibrosis in mice by inhibiting the secretion of interleukin-17A. Acta Pharmacol Sin. (2022) 43:908–18. doi: 10.1038/s41401-021-00706-4, PMID: 34316030 PMC8976043

[12] King TE Jr Bradford WZ Castro-Bernardini S Fagan EA Glaspole I Glassberg MK. A phase 3 trial of pirfenidone in patients with idiopathic pulmonary fibrosis. N Engl J Med. (2014) 370:2083–92. doi: 10.1056/NEJMoa1402582, PMID: 24836312

[13] Noble PW Albera C Bradford WZ Costabel U Glassberg MK Kardatzke D. Pirfenidone in patients with idiopathic pulmonary fibrosis (CAPACITY): two randomised trials. Lancet. (2011) 377:1760–9. doi: 10.1016/s0140-6736(11)60405-4, PMID: 21571362

[14] Nogales C Mamdouh ZM List M Kiel C Casas AI Schmidt H. Network pharmacology: curing causal mechanisms instead of treating symptoms. Trends Pharmacol Sci. (2022) 43:136–50. doi: 10.1016/j.tips.2021.11.004, PMID: 34895945

[15] Lv Q Wang J Xu C Huang X Ruan Z Dai Y. Pirfenidone alleviates pulmonary fibrosis in vitro and in vivo through regulating Wnt/GSK-3β/β-catenin and TGF-β1/Smad2/3 signaling pathways. Mol Med. (2020) 26:49. doi: 10.1186/s10020-020-00173-3, PMID: 32448163 PMC7245944

[16] Ballester B Milara J Cortijo J. Pirfenidone anti-fibrotic effects are partially mediated by the inhibition of MUC1 bioactivation. Oncotarget. (2020) 11:1306–20. doi: 10.18632/oncotarget.27526, PMID: 32341751 PMC7170494

[17] Zhang W Zhou H Jiang Y He J Yao Y Wang J. Acinetobacter baumannii outer membrane protein a induces pulmonary epithelial barrier dysfunction and bacterial translocation through the TLR2/IQGAP1 Axis. Front Immunol. (2022) 13:927955. doi: 10.3389/fimmu.2022.927955, PMID: 35844614 PMC9280087

[18] Zhen Z Xia L You H Jingwei Z Shasha Y Xinyi W. An integrated gut microbiota and network pharmacology study on Fuzi-Lizhong pill for treating diarrhea-predominant irritable bowel syndrome. Front Pharmacol. (2021) 12:746923. doi: 10.3389/fphar.2021.746923, PMID: 34916934 PMC8670173

[19] Rajpoot S Solanki K Kumar A Zhang KYJ Pullamsetti SS Savai R. In-silico Design of a Novel Tridecapeptide Targeting Spike Protein of SARS-CoV-2 variants of concern. Int J Pept Res Ther. (2022) 28:28. doi: 10.1007/s10989-021-10339-0, PMID: 34924897 PMC8667532

[20] Al-Roub A Akhter N Al-Rashed F Wilson A Alzaid F Al-Mulla F. TNFα induces matrix metalloproteinase-9 expression in monocytic cells through ACSL1/JNK/ERK/NF-kB signaling pathways. Sci Rep. (2023) 13:14351. doi: 10.1038/s41598-023-41514-6, PMID: 37658104 PMC10474281

[21] Capece D Verzella D Flati I Arboretto P Cornice J Franzoso G. NF-κB: blending metabolism, immunity, and inflammation. Trends Immunol. (2022) 43:757–75. doi: 10.1016/j.it.2022.07.004, PMID: 35965153

[22] Colleselli K Stierschneider A Wiesner C. An update on toll-like receptor 2, its function and dimerization in pro- and anti-inflammatory processes. Int J Mol Sci. (2023) 24:12464. doi: 10.3390/ijms241512464, PMID: 37569837 PMC10419760

[23] Hou A Tin MQ Tong L. Toll-like receptor 2-mediated NF-kappa B pathway activation in ocular surface epithelial cells. Eye Vis (Lond). (2017) 4:17. doi: 10.1186/s40662-017-0082-x, PMID: 28706958 PMC5506675

[24] Guo Q Jin Y Chen X Ye X Shen X Lin M. NF-κB in biology and targeted therapy: new insights and translational implications. Signal Transduct Target Ther. (2024) 9:53. doi: 10.1038/s41392-024-01757-9, PMID: 38433280 PMC10910037

[25] Dutta D Jana M Majumder M Mondal S Roy A Pahan K. Selective targeting of the TLR2/MyD88/NF-κB pathway reduces α-synuclein spreading in vitro and in vivo. Nat Commun. (2021) 12:5382. doi: 10.1038/s41467-021-25767-1, PMID: 34508096 PMC8433339

[26] Antar SA Saleh MA Al-Karmalawy AA. Investigating the possible mechanisms of pirfenidone to be targeted as a promising anti-inflammatory, anti-fibrotic, anti-oxidant, anti-apoptotic, anti-tumor, and/or anti-SARS-CoV-2. Life Sci. (2022) 309:121048. doi: 10.1016/j.lfs.2022.121048, PMID: 36209833 PMC9536875

[27] Bai X Nie P Lou Y Zhu Y Jiang S Li B. Pirfenidone is a renal protective drug: mechanisms, signalling pathways, and preclinical evidence. Eur J Pharmacol. (2021) 911:174503. doi: 10.1016/j.ejphar.2021.174503, PMID: 34547247

[28] Ruwanpura SM Thomas BJ Bardin PG. Pirfenidone: molecular mechanisms and potential clinical applications in lung disease. Am J Respir Cell Mol Biol. (2020) 62:413–22. doi: 10.1165/rcmb.2019-0328TR, PMID: 31967851

[29] Alim LF Keane C Souza-Fonseca-Guimaraes F. Molecular mechanisms of tumour necrosis factor signalling via TNF receptor 1 and TNF receptor 2 in the tumour microenvironment. Curr Opin Immunol. (2024) 86:102409. doi: 10.1016/j.coi.2023.102409, PMID: 38154421

[30] Liu TT Sun HF Han YX Zhan Y Jiang JD. The role of inflammation in silicosis. Front Pharmacol. (2024) 15:1362509. doi: 10.3389/fphar.2024.1362509, PMID: 38515835 PMC10955140

[31] Zhang M Peng LL Ji XL Yang HB Zha RS Gui GP. Tumor necrosis factor gene polymorphisms are associated with silicosis: a systemic review and meta-analysis. Biosci Rep. (2019) 39:BSR20181896. doi: 10.1042/bsr20181896, PMID: 30643011 PMC6361771

[32] Jiang PR Cao Z Qiu ZL Pan JW Zhang N Wu YF. Plasma levels of TNF-α and MMP-9 in patients with silicosis. Eur Rev Med Pharmacol Sci. (2015) 19:1716–20. PMID: 26004615

[33] Su L Dong Y Wang Y Wang Y Guan B Lu Y. Potential role of senescent macrophages in radiation-induced pulmonary fibrosis. Cell Death Dis. (2021) 12:527. doi: 10.1038/s41419-021-03811-8, PMID: 34023858 PMC8141056

[34] Long L Dai X Yao T Zhang X Jiang G Cheng X. Mefunidone alleviates silica-induced inflammation and fibrosis by inhibiting the TLR4-NF-κB/MAPK pathway and attenuating pyroptosis in murine macrophages. Biomed Pharmacother. (2024) 178:117216. doi: 10.1016/j.biopha.2024.117216, PMID: 39096618

[35] Wang Y Jiao L Qiang C Chen C Shen Z Ding F. The role of matrix metalloproteinase 9 in fibrosis diseases and its molecular mechanisms. Biomed Pharmacother. (2024) 171:116116. doi: 10.1016/j.biopha.2023.116116, PMID: 38181715

[36] Kumari S Singh P Singh R. Repeated silica exposures lead to silicosis severity via PINK1/PARKIN mediated mitochondrial dysfunction in mice model. Cell Signal. (2024) 121:111272. doi: 10.1016/j.cellsig.2024.111272, PMID: 38944258

[37] Craig VJ Zhang L Hagood JS Owen CA. Matrix metalloproteinases as therapeutic targets for idiopathic pulmonary fibrosis. Am J Respir Cell Mol Biol. (2015) 53:585–600. doi: 10.1165/rcmb.2015-0020TR, PMID: 26121236 PMC4742954

[38] Zheng M Karki R Williams EP Yang D Fitzpatrick E Vogel P. TLR2 senses the SARS-CoV-2 envelope protein to produce inflammatory cytokines. Nat Immunol. (2021) 22:829–38. doi: 10.1038/s41590-021-00937-x, PMID: 33963333 PMC8882317

[39] Li C Liu M Deng L Luo D Ma R Lu Q. Oxyberberine ameliorates TNBS-induced colitis in rats through suppressing inflammation and oxidative stress via Keap1/Nrf2/NF-κB signaling pathways. Phytomedicine. (2023) 116:154899. doi: 10.1016/j.phymed.2023.154899, PMID: 37247589

[40] Peng L Wen L Shi QF Gao F Huang B Meng J. Scutellarin ameliorates pulmonary fibrosis through inhibiting NF-κB/NLRP3-mediated epithelial-mesenchymal transition and inflammation. Cell Death Dis. (2020) 11:978. doi: 10.1038/s41419-020-03178-2, PMID: 33188176 PMC7666141

[41] Tian Y Shi H Zhang D Wang C Zhao F Li L. Nebulized inhalation of LPAE-HDAC10 inhibits acetylation-mediated ROS/NF-κB pathway for silicosis treatment. J Control Release. (2023) 364:618–31. doi: 10.1016/j.jconrel.2023.10.018, PMID: 37848136

[42] Gottschalk I Kölsch U Wagner DL Kath J Martini S Krüger R. IRAK1 duplication in MECP2 duplication syndrome does not increase canonical NF-κB-induced inflammation. J Clin Immunol. (2023) 43:421–39. doi: 10.1007/s10875-022-01390-7, PMID: 36319802 PMC9628328

[43] Di Giuseppe M Gambelli F Hoyle GW Lungarella G Studer SM Richards T. Systemic inhibition of NF-kappaB activation protects from silicosis. PLoS One. (2009) 4:e5689. doi: 10.1371/journal.pone.0005689, PMID: 19479048 PMC2682759

[44] Tan S Chen S. Macrophage autophagy and silicosis: current perspective and latest insights. Int J Mol Sci. (2021) 22:453. doi: 10.3390/ijms22010453, PMID: 33466366 PMC7795780

[45] Guo L Han L Zhang J Shen M Li J Zhang K. HMGB1 mediates epithelial-mesenchymal transition and fibrosis in silicosis via RAGE/β-catenin signaling. Chem Biol Interact. (2025) 408:111385. doi: 10.1016/j.cbi.2025.111385, PMID: 39800143

[46] Zhou Q Yi G Chang M Li N Bai Y Li H. Activation of Sirtuin3 by honokiol ameliorates alveolar epithelial cell senescence in experimental silicosis via the cGAS-STING pathway. Redox Biol. (2024) 74:103224. doi: 10.1016/j.redox.2024.103224, PMID: 38865904 PMC11215422

